# Taking two to tango: a role for ghrelin receptor heterodimerization in stress and reward

**DOI:** 10.3389/fnins.2013.00148

**Published:** 2013-08-30

**Authors:** Harriët Schellekens, Timothy G. Dinan, John F. Cryan

**Affiliations:** ^1^Food for Health Ireland, University College CorkCork, Ireland; ^2^Laboratory of Neurogastroenterology, Alimentary Pharmabiotic Centre, University College CorkCork, Ireland; ^3^Department of Psychiatry, University College CorkCork, Ireland; ^4^Department of Anatomy and Neuroscience, University College CorkCork, Ireland

**Keywords:** ghrelin, dimerization, obesity, stress, food reward

## Abstract

The gut hormone, ghrelin, is the only known peripherally derived orexigenic signal. It activates its centrally expressed receptor, the growth hormone secretagogue receptor (GHS-R1a), to stimulate food intake. The ghrelin signaling system has recently been suggested to play a key role at the interface of homeostatic control of appetite and the hedonic aspects of food intake, as a critical role for ghrelin in dopaminergic mesolimbic circuits involved in reward signaling has emerged. Moreover, enhanced plasma ghrelin levels are associated with conditions of physiological stress, which may underline the drive to eat calorie-dense “comfort-foods” and signifies a role for ghrelin in stress-induced food reward behaviors. These complex and diverse functionalities of the ghrelinergic system are not yet fully elucidated and likely involve crosstalk with additional signaling systems. Interestingly, accumulating data over the last few years has shown the GHS-R1a receptor to dimerize with several additional G-protein coupled receptors (GPCRs) involved in appetite signaling and reward, including the GHS-R1b receptor, the melanocortin 3 receptor (MC_3_), dopamine receptors (D_1_ and D_2_), and more recently, the serotonin 2C receptor (5-HT_2C_). GHS-R1a dimerization was shown to affect downstream signaling and receptor trafficking suggesting a potential novel mechanism for fine-tuning GHS-R1a receptor mediated activity. This review summarizes ghrelin's role in food reward and stress and outlines the GHS-R1a dimer pairs identified to date. In addition, the downstream signaling and potential functional consequences of dimerization of the GHS-R1a receptor in appetite and stress-induced food reward behavior are discussed. The existence of multiple GHS-R1a heterodimers has important consequences for future pharmacotherapies as it significantly increases the pharmacological diversity of the GHS-R1a receptor and has the potential to enhance specificity of novel ghrelin-targeted drugs.

## Introduction

Appetite regulation, food intake and diet are closely intertwined with mood regulation and stress perception and stress response (Oliver and Wardle, 1999; Gibson, 2006; Morrison, 2009; Dallman, 2010). Obesity and the metabolic syndrome, which can be defined as a combination of comorbid medical disorders, including atherosclerosis, hypertension, insulin resistance or diabetes mellitus type II, glucose intolerance, dyslipidemia, and a general pro-inflammatory phenotype (Cheng and Leiter, 2006; Mikhail, 2009), have been identified as environmental risk factors for affective psychiatric disorders, including anxiety and depression (McElroy et al., 2004; Goldbacher and Matthews, 2007; Kloiber et al., 2007; Gariepy et al., 2010; Marijnissen et al., 2011). Epidemiologic data even suggests that obesity is associated with a 25% increased incidence of anxiety and mood disorders (Simon et al., 2006). In addition, major depression in adolescence is linked with a higher risk for obesity in adulthood (Richardson et al., 2003). Moreover, metabolic conditions may be exacerbated in depression and *vice versa*, which indicates a reciprocal link (McElroy et al., 2004; Simon et al., 2006; de Wit et al., 2010; Luppino et al., 2010; Marijnissen et al., 2011). Likewise, stress significantly impacts on food intake in humans and animals and may promote metabolic disturbances (Block et al., 2009; Dallman, 2010; Maniam and Morris, 2012). Interestingly, stress-induced hyperphagia and subsequent increases in body weight and obesity are also associated with major depressive disorders in humans (Novick et al., 2005; Simon et al., 2006; Kloiber et al., 2007). Moreover, acute stress responses are reduced following intake of palatable rewarding foods, potentially explaining the phenomenon of “comfort eating” observed in stressed individuals as self-medication for stress relief (Dallman et al., 2003).

Ghrelin, a 28 amino acid stomach-derived peptide (Kojima et al., 1999), is currently the only described orexigenic hormone from the periphery, acting centrally to increase food intake and modulates the body's metabolism via centrally activated mechanisms (Tschop et al., 2000; Nakazato et al., 2001; Kojima et al., 2004; Andrews, 2011). Peripheral ghrelin mediates its appetite-inducing effects centrally, after passing through the blood brain barrier (Banks et al., 2002, 2008). Recent experiments by Schaeffer and colleagues have confirmed that the hypothalamus directly senses circulating ghrelin from the periphery to modify energy status (Schaeffer et al., 2013). This study demonstrated that circulating ghrelin rapidly binds neurons in the vicinity of fenestrated capillaries using *in vivo* multiphoton microscopy together with fluorescently labeled ligands. Interestingly, it was also shown that the number of labeled cell bodies varies with feeding status, suggesting a potential differential ability to sense ghrelin under altered metabolic conditions. In addition, in both rodents and humans, ghrelin also reaches the brain via vagal afferents to the nucleus of the solitary tract (NTS) in the brain stem with further projections to the arcuate nucleus (ARC) of the hypothalamus (Asakawa et al., 2001b; Date et al., 2002; Williams et al., 2003; le Roux et al., 2005). However, contradictory results exists in rats, disputing the role of vagal afferents in the acute eating-stimulatory effect of peripheral ghrelin (Arnold et al., 2006).

When the orexigenic hormone ghrelin activates its receptor, the growth-hormone secretagogue receptor (GHS-R1a), it stimulates appetite and food intake but also mediates a multitude of additional biological activities, including the secretion of growth hormone (GH), glucose and lipid metabolism and gastrointestinal motility, which together maintain the body's energy homeostasis (for review see Schellekens et al., 2010). Moreover, ghrelin contributes to the accumulation of adipose tissue via the promotion of carbohydrates over fat as energy substrate (Tschop et al., 2000). The ghrelinergic system has therefore received considerable attention in the pharmaceutical industry as a promising target in obesity treatment and other eating disorders (Horvath et al., 2003; Zorrilla et al., 2006; Leite-Moreira and Soares, 2007; Moulin et al., 2007; Soares et al., 2008; Chollet et al., 2009; Lu et al., 2009; Schellekens et al., 2010; Patterson et al., 2011; Yi et al., 2011). In addition, accumulating data has revealed that the ghrelinergic system has an important function in other behaviors related to food intake and plays a pivotal role in the mesolimbic dopaminergic circuitry, which is responsible for various non-homeostatic, hedonic rewarding and motivational aspects of food intake (for review see Dickson et al., 2011; Egecioglu et al., 2011; Skibicka and Dickson, 2011; Perello and Zigman, 2012; Schellekens et al., 2012b; Skibicka et al., 2012). More recently, ghrelin has been shown to be involved in mediating a stress response and to mediate stress-induced food reward behavior (Lutter et al., 2008; Chuang and Zigman, 2010; Patterson et al., 2010, 2013; Chuang et al., 2011; Diz-Chaves, 2011; Schellekens et al., 2012b; Spencer et al., 2012).

Thus, current research indicates a potential link between ghrelin and affective disorders, such as anxiety and depression. In line with this hypothesis are the decreased plasma ghrelin levels often observed in depressed patients (Barim et al., 2009) (but also see Schanze et al., 2008; Kluge et al., 2009). Furthermore, recent data also demonstrates that ghrelin administration in patients with major depression has some antidepressant effects (Kluge et al., 2011), which is in support of the involvement of ghrelin in the etiology of depressive disorders. Interestingly, a ghrelin gene polymorphism has also been linked with the symptomatology of depression (Nakashima et al., 2008). It is clear, that the ghrelin signaling system is involved in a multitude of centrally regulated functionalities, which exceeds far beyond appetite regulation, energy homeostasis and GH secretion. This is reinforced by the ubiquitous expression of the GHS-R1a receptor in both the periphery as well as the brain (Howard et al., 1996; Guan et al., 1997; Zigman et al., 2006). In particular, the extra-hypothalamic expression of the GHS-R1a receptor, including in the ventral tegmental area (VTA) and nucleus accumbens (NAcc), hippocampus and amygdala, reinforces a function for ghrelin signaling in the hedonic regulation of food intake (Guan et al., 1997; Zigman et al., 2006). Together, this data suggests that the role of the ghrelinergic system in obesity, appetite and food intake now extends toward the reward and motivation pathways as well as to the signaling pathways involved in stress, psychiatric disposition (i.e., mood) and affective disorders such as anxiety and depression. However, it is currently unclear what the exact molecular mechanisms are that mediate this biological diversity of the GHS-R1a receptor. We hypothesize that the differential GHS-R1a receptor signaling events can be explained through crosstalk with additional neuropeptide systems and dimerization with other G-protein coupled receptors (GPCRs) (Schellekens et al., 2013a,b).

**Figure 1:**
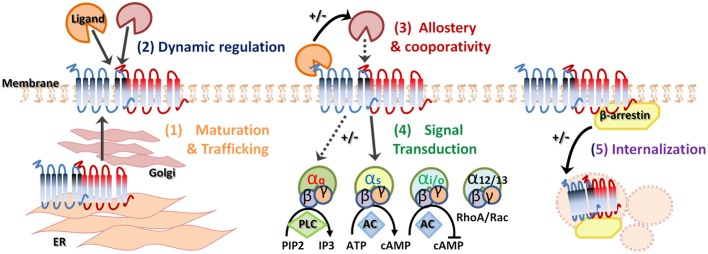
**Heterodimerization of G-protein coupled receptors.** G-protein coupled receptor (GPCR) oligomerization has several biological functions and consequences. Receptor dimerization can play a role in receptor maturation and correct trafficking **(1)**. Specific ligand binding can dynamically regulate heterodimerization **(2)** and allostery can enhance or suppress downstream signaling **(3)**. In addition, GPCR heterodimerization may demonstrate preferential G protein coupling **(4)**. Finally, agonist-promoted GPCR endocytosis and co-internalization may lead to signal attenuation **(5)**. +/− indicates increase or decrease, respectively.

This review outlines ghrelin's role in food reward signaling and stress, highlights the identified GHS-R1a dimer pairs to date, and discusses the potential functional consequences of dimerization of the GHS-R1a receptor, with particular focus on its key role in appetite and stress-induced food reward behavior. The ability of the GHS-R1a receptor to form heterodimers with multiple GPCRs involved in the homeostatic or hedonic regulation of food intake and potentially also in the stress response significantly increases its pharmacological diversity (Figure 1). Further understanding of the biological significance of GHS-R1a receptor dimers in the neuroendocrine system may pertain to potential new molecular targets and novel strategies for the treatment of stress-associated psychiatric disorders of anxiety as well as eating disorders and metabolic disturbances leading to obesity.

## Ghrelin signaling in stress and reward

The hedonic signaling following ingestion of palatable and calorie-rich food is increasingly being recognized as an important underlying cause for the increase in obesity worldwide, as the overconsumption of calorie-dense foods extends far beyond the individual's nutritional needs (Berthoud, 2006). Obesity-associated habitual overconsumption is mediated by rewarding feelings associated with eating as well as by a natural sensitivity to food stimuli. Stress also affects feeding behavior in humans (Oliver and Wardle, 1999; Gibson, 2006; Dallman, 2010). Interestingly, while some individuals display a stress-induced hyperphagia, others display hypophagia, but an overall increased consumption of highly palatable and caloric-dense foods compared to non-stressed controls is reported (Gibson, 2006; Dallman, 2010). Stress-related psychiatric disorders, including depression, are also linked with extremes in eating behavior according to criteria within the diagnostic and statistical manual of mental disorders (DSM-IV) (American Psychiatric Association, 1994). In addition, the experience of intense stress during childhood represents a strong risk factor to develop depression later in life (O'Mahony et al., 2009; Heim and Binder, 2012). Moreover, these same early life stress events have been associated with metabolic abnormalities later in life (Kaufman et al., 2007). The complex bi-directional relationship between stress, mood and feeding behavior are regulated by converging neuronal pathways mediating appetite and subsequent food intake and circuitries that modulate stress via the hypothalamic-pituitary-adrenal (HPA) axis (Kyrou and Tsigos, 2007; Ulrich-Lai and Herman, 2009; Dallman, 2010; Finger et al., 2011, 2012b; Maniam and Morris, 2012; Schellekens et al., 2012b; Scott et al., 2012). The current knowledge on the role of the metabolic peptide ghrelin in this non-homeostatic food intake behavior is discussed below.

### Feeding behavior and stress response

Stress response in mammals is mediated via the sympathetic nervous system and involves activation of the HPA axis, immunological changes as well as changes in neural and endocrine mechanisms (McEwen, 2007; Ulrich-Lai and Herman, 2009). The behavioral and physiological responses following stress are initiated with the release of corticotrophin-releasing factor (CRF) from specialized neurons in the paraventricular nucleus (PVN) of the hypothalamus, which subsequently induces the release of adrenocorticotropic hormone (ACTH) from the anterior pituitary gland into the general circulation. This ultimately stimulates the release of the glucocorticoid corticosterone (or cortisol in primates) from the adrenal cortex. A negative feedback loop exists, which is activated after the elevation in circulating glucocorticoids and prevents further stimulation of the HPA axis via activation of glucocorticoid receptors expressed on the hypothalamus, hippocampus, medial prefrontal cortex, and pituitary gland (Jones et al., 1977; Mahmoud et al., 1984; Sapolsky et al., 1984, 2000; Diorio et al., 1993; Radley et al., 2008; Ulrich-Lai and Herman, 2009). The acute activation of the HPA axis following stress promotes survival via physiological and behavioral adaptations and aims to re-establish the challenged body equilibrium. However, exposure to chronic stress and overstimulation of the HPA-axis can result in physiological alterations with detrimental effects and metabolic disturbances, including obesity (Black, 2006; McEwen, 2007). *Vice versa*, obesity constitutes a chronic stressful state that may cause HPA axis dysfunction (Kyrou and Tsigos, 2007). Interestingly, and in line with the above, the HPA axis has been linked with the control of metabolism and neurotransmitter release in several brain regions, facilitating the appropriate channeling of energy resources to promote survival following stress (Kyrou and Tsigos, 2007; McEwen, 2007; Dallman, 2010; Bowers et al., 2012).

It is likely that the neurobiological mechanisms at the heart of the relationship between food intake and stress responses and the neuronal circuitry of fear, underlying anxiety, are evolutionary selected to function as defensive survival mechanism regulating our response to environmental threats (Siervo et al., 2009). The link between stress and feeding behavior is reinforced by the overlapping neuronal circuitry between the two and involves the same neuropeptides (Bowers et al., 2012; Maniam and Morris, 2012). The stress response following HPA axis activation, and the central mechanisms that regulate appetite and subsequent food intake, proopiomelanocortin (POMC) or neuropeptide Y and agouti-related peptide (NPY/AgRP), both converge on the PVN and on the CRF-producing neurons within this region. In addition, several peripherally derived hormones and neuropeptides regulating appetite and satiety also play a key role in anxiety-like behavior, highlighting the importance of a bi-directional relationship in anxiety-related mechanisms and eating disorders. For example, the orexigenic neuropeptide NPY (Stanley and Leibowitz, 1985; Heilig et al., 1989) has been shown to exert anxiolytic effects and the anorexigenic peptide cholecystokinin (CCK) (Gibbs et al., 1973) induces panic-like effects (Strohle et al., 2000), impacting on general anxiety and stress-related behavior. Moreover, cocaine- and amphetamine-regulated transcript (CART) is both satiety inducing as well as a mediator of anxiety-like behaviors (Kristensen et al., 1998; Stanek, 2006) and neuropeptide S has both anorexigenic (Smith et al., 2006) and anxiolytic (Xu et al., 2004) responses. Furthermore, neuropeptide expression has been shown to be altered following acute and chronic stressors (Bowers et al., 2012). Likewise, the CRF system is implicated in the regulation of energy balance and in the pathophysiology of obesity and eating disorders. The corticostriatal-hypothalamic circuitry mediates the motivation to obtain food rewards and to promote the overconsumption of palatable foods beyond acute homeostatic needs (Kelley et al., 2005). However, the precise effects of chronic psychosocial stress on neuropeptide and gut hormone expression under conditions of obesity as well as under conditions of malnutrition or anorexia nervosa have not been extensively explored. However, as most neuropeptides convey onto centrally expressed GPCRs, we hypothesize a crucial role for GPCR dimerization and oligomerization.

### Ghrelin signaling in stress

Recently, a key role for ghrelin in anxiety and in stress-induced food intake behavior has been suggested (Chuang and Zigman, 2010; Chuang et al., 2011; Schellekens et al., 2012b; Spencer et al., 2012; Patterson et al., 2013). Preproghrelin mRNA levels have been shown to be up-regulated in conditions of stress in both rodents and humans (Asakawa et al., 2001a; Kristenssson et al., 2006; Rouach et al., 2007; Ochi et al., 2008). In addition, circulating levels of plasma ghrelin increase in parallel with corticosterone following both acute and chronic stressors and remain elevated after cessation of the stressor (Asakawa et al., 2001a; Kristenssson et al., 2006; Ochi et al., 2008; Zheng et al., 2009). This may suggest that ghrelin functions as a potential defence mechanism against the consequences of stress and to prevent the manifestation of anxiety and depressive-like symptoms following chronic stress (Lutter et al., 2008). In line with this hypothesis was the observed ghrelin-mediated activation of CRF-producing neurons and enhanced CRF gene expression in the PVN of the hypothalamus (Cabral et al., 2012). In addition, recent findings suggest that CRF type 2 receptors may mediate some metabolic actions of ghrelin (Gershon and Vale, 2013). In this study, a CRF type 2 receptor selective antagonist, anti-sauvagine-30, blocked the ghrelin-mediated increase of glucose uptake as well as the ghrelin-induced enhanced expression of mitochondrial uncoupling proteins 2 and 3 (UCP2 and UCP3) in C2C12 cells, a mouse myoblast cell line. Moreover, both female and male cortistatin (CST) knockout mice were associated with enhanced levels of corticosterone compared to control as well as enhanced ACTH levels in female CST knockout mice. Furthermore, the observed stimulatory effects on prolactin secretion in the CST knockout mice were blocked by a GHS-R1a receptor antagonists and CST knockouts were also correlated with an enhanced level of circulating acyl-ghrelin and increased expression of stomach ghrelin O-acyl transferase (GOAT) (Cordoba-Chacon et al., 2011). The neuropeptide CST is able to bind the GHS-R1a receptor and these findings may further suggest a potential role for CST and ghrelin in the dysregulation of the HPA axis.

Interestingly, ghrelin knockout mice have been found to be more anxious in behavioral tests after acute restraint stress compared with wild-type mice but exhibited a reduced ACTH response and a lower corticosterone response, suggesting that in absence of ghrelin the glucocorticoid negative feedback may be dysregulated (Spencer et al., 2012). Thus, ghrelin may act to reduce anxiety following an acute stressor by stimulating the HPA axis at the level of the anterior pituitary and releasing glucocorticoids (Spencer et al., 2012). Interestingly, glucocorticoids have also been shown to increase the motivation for certain foods (Dallman et al., 2006; Zimmermann et al., 2007; Kageyama et al., 2012) as well as to further activate the ghrelinergic system (Kageyama et al., 2012), reinforcing the feedback theory between chronic stress and food intake and the involvement of ghrelin. Together, we can conclude that under normal physiology ghrelin may contribute to the stress-induced rise in glucocorticoids, activating the negative feedback loop in an attempt to prevent HPA-axis overstimulation.

In line with these findings, increases in ghrelin levels following subcutaneous injections or calorie restriction produced anxiolytic- and antidepressant-like responses in the elevated plus maze and forced swim test (Lutter et al., 2008). In the same study, chronic social defeat stress (CSDS), a model of prolonged psychosocial stress, enhanced acylated ghrelin levels in mice. Moreover, social avoidance was increased in GHS-R1a null mice, suggesting ghrelin to regulate social isolation in response to CSDS (Lutter et al., 2008). Another study, showed a decreased immobility of mice in the forced swim test and increased time spent in the open arms of the elevated plus maze following caloric restriction or subcutaneous ghrelin injection, reinforcing the anxiolytic and antidepressant-like effects of elevated plasma ghrelin (Patterson et al., 2010). In contrast, enhanced ghrelin levels have also been implicated in the induction of anxiety-like and depressive-like behavior (Asakawa et al., 2001a; Carlini et al., 2002, 2004; Kanehisa et al., 2006). These opposing findings are not easily reconciled but have been attributed to the type and duration of stressor used (Stengel et al., 2011) and to differential experimental conditions (for review see Chuang and Zigman, 2010). In these studies, different types of stress yielded differential circulating levels of ghrelin, (Stengel et al., 2011). Circulating ghrelin was elevated following metabolic stressors, including caloric restriction, acute fasting as well as cold exposure, and psychological stressors, such as unpredictable or CSDS. Ghrelin secretion following these stressors is suggested to be mediated via a sympathoadrenal response after sympathetic nervous system activation and catecholamine release (Mundinger et al., 2006; Zhao et al., 2010). However, physical stressors, such as immunological/endotoxin injection, abdominal surgery and exercise reduced plasma ghrelin levels. Further research is needed to delineate the divergent pathways underlying the changes in circulating ghrelin levels following differential stressors.

Only a few clinical studies are available on ghrelin's role in stress, anxiety and depression. Despite the limited number of human studies reported, these also have found that administration of ghrelin induces cortisol and ACTH secretion (Takaya et al., 2000; Arvat et al., 2001). Moreover, elevated circulating plasma ghrelin levels are equally observed in humans in response to stress exposure, which were found to correlate with cortisol levels following the standardized Trier-Social-Stress test (Rouach et al., 2007). In this study, stress perception and anxiety were enhanced and plasma serum cortisol levels were acutely increased. Interestingly, cortisol levels correlated with an increase in ghrelin but not to BMI or eating scores. Interestingly, while in non-emotional eaters baseline ghrelin exceeded that of high emotional eaters, ghrelin levels declined in the low emotional eaters following food intake, but not in emotional eaters, which may explain sustained eating in high emotional eaters (Raspopow et al., 2010). Moreover, ghrelin is able to induce an exaggerated ACTH response, independent of ghrelin-mediated GH response in Cushing's disease, a disorder characterized by major weight gain and chronic hypercortisolism (Monsonego et al., 2001; Moon et al., 2011). Interestingly, monoaminergic neurotransmitters are both activated by stressors and modulated by ghrelin (Brunetti et al., 2002; Date et al., 2006; Nonogaki et al., 2006; Kawakami et al., 2008). For example, dopaminergic cells in the VTA are sensitive to ghrelin, as well as noradrenergic cells in the NTS in the brainstem and serotinergic cells in the midbrain raphe nuclei (Carlini et al., 2004; Abizaid et al., 2006; Date et al., 2006). In addition, these ghrelin-sensitive monoamines mediate the activity of limbic and hypothalamic structures associated with stress-associated dysfunction (Anisman and Zacharko, 1990; Bremner et al., 1996). This ghrelin-induced activity may be mediated via specific GHS-R1a heterodimers present on these monoaminergic cells.

### Ghrelin signaling in food reward behavior

Ghrelin can promote the predisposition to overeat when presented with pleasurable energy dense food sources, highlighting the key role for ghrelin signaling in the motivation to obtain palatable food rewards (for review see Skibicka and Dickson, 2011; Perello and Zigman, 2012; Schellekens et al., 2012b). Indeed, both peripheral and central ghrelin were shown to enhance hedonic feeding associated with food palatability (Shimbara et al., 2004; Disse et al., 2010). The expression of the GHS-R1a receptor on midbrain dopaminergic neurons in the VTA and NAcc is in line with ghrelin's role in hedonic eating behavior mediated in the mesolimbic reward system (Guan et al., 1997; Abizaid, 2009; Skibicka et al., 2011). Indeed, administration of ghrelin into the VTA and NAcc of rats directly activated these regions and stimulated chow hyperphagia (Naleid et al., 2005). In addition, direct injection of ghrelin into the VTA of mice significantly increased the preference for rewarding foods (Egecioglu et al., 2010).

The ghrelin-mediated enhanced preference for rewarding foods was absent in GHS-R1a knock-out mice (Disse et al., 2010) and following GHS-R1a antagonist treatment in rats (Egecioglu et al., 2010), demonstrating GHS-R1a receptor dependence. In addition, several studies, using conditioned place preference (CPP) in rodents, have demonstrated that increases in ghrelin, following peripheral or central administration and caloric restriction, enhance the CPP response for HFD but not chow (Chuang and Zigman, 2010; Egecioglu et al., 2010; Perello et al., 2010; Disse et al., 2011). Moreover, this was dependent on the GHS-R1a receptor and an intact VTA region. Furthermore, the ability of ghrelin to alter food-associated reward has been assessed using operant conditioning paradigms (Perello et al., 2010; Skibicka et al., 2011, 2012; Finger et al., 2012a). In these studies, intra-VTA microinjection of ghrelin significantly stimulated free feeding of chow and increased operant responding to palatable rewards in rodents (Skibicka et al., 2011), while this did not occur when ghrelin was directly microinjected into the NAcc (Dickson et al., 2011; Skibicka et al., 2011). Interestingly, these studies also demonstrated that ghrelin enhances incentive motivation for sucrose rewards in satiated rats but when ghrelin signaling is blocked operant responding for sugar in hungry rats returns to the level of satiated rats (Skibicka et al., 2012). Moreover, GHS-R1a blockade in the VTA decreased the motivation to obtain sucrose rewards but did not affect fasting induced chow hyperphagia (Skibicka et al., 2011). This suggests the selection of rewarding foods and food-motivated behavior is attributed more specifically to ghrelin's action on the VTA and not the NAcc.

Finally, data is accumulating identifying the ghrelinergic system to mediate food reward following exposure to stress (Asakawa et al., 2001a; Chuang and Zigman, 2010; Chuang et al., 2011; Schellekens et al., 2012b; Spencer et al., 2012; Patterson et al., 2013). For example, intake of high-fat diet and the CPP response are increased after exposure to CSDS and absent in GHS-R1a knockout mice, demonstrating again dependence on intact ghrelin signaling (Chuang et al., 2011).

Ghrelin's ability to modify the rewarding properties of palatable foods is mediated via connections to dopamine (DA) neurons and DA release from mesolimbic dopaminergic neurons in the VTA, which project to the NAcc (Jerlhag et al., 2007; Dickson et al., 2011; Skibicka and Dickson, 2011). The direct activation of dopaminergic neurons in the VTA following peripheral and central ghrelin administration is in support of this notion (Abizaid et al., 2006). Moreover, direct injection of ghrelin in the VTA increases appetitive motivation in rats as shown by an increase in breakpoint in a progressive ratio schedule and this was shown to be dependent on intact dopamine signaling (Weinberg et al., 2011). The ghrelin-mediated increase in food-reinforced behavior was absent and breakpoints were stabilized following administration of the dopaminergic neurotoxin 6-hydroxydopamine (6-OHDA), which significantly depleted striatal dopamine. These findings demonstrate that activation of dopamine neurons is crucial for the ghrelin-mediated potentiation of food reward in the mesolimbic reward pathway.

We suggest the involvement of specific heterodimers of the GHS-R1a receptor with dopamine D_1_ and D_2_ receptors, which are involved in the rewarding aspects of food. These specific GHS-R1a dimers may mediate ghrelin's function in the dopaminergic reward-signaling pathway within the VTA. Indeed, evidence for heterodimerization of the GHS-R1a and D_1_ and D_2_ receptor was recently demonstrated (Jiang et al., 2006; Kern et al., 2012) and is further discussed in this review in the next paragraphs.

### Potential mechanism of stress-induced ghrelin signaling in food reward behavior

It is clear that stress perception and response are modulated by food intake and diet (Oliver and Wardle, 1999; Gibson, 2006; Morrison, 2009; Dallman, 2010). The hormone ghrelin is poised to be involved in the association between stress, food intake and diet, considering the strong impact of ghrelin on both physiological processes. Interestingly, typical stress-induced metabolic changes in caloric intake, caloric efficiency and body-weight gain are absent in GHS-R1a null mice, suggesting that ghrelin is important for the metabolic shift required to deal with the energetic challenge of chronic stress (Patterson et al., 2010). Moreover, the stress-induced rise in circulating ghrelin may be behind the phenomenon of “comfort eating” observed in conditions of stress. This hypothesis is in line with the suggested function of ghrelin as an energy deficit signal, which may have evolved to favor consumption of calorie-dense palatable foods and to protect the storage of fat in times of energy insufficiency (Wells, 2009). Indeed, a continuous peripheral or central ghrelin infusion decreases fat utilization as a fuel substrate without significantly changing energy expenditure or locomotor activity, ultimately leading to increased adiposity (Tschop et al., 2000). This may be beneficial under conditions of stress as fat stores are maintained while energy from carbohydrates is available as a rapid fuel source, to maintain the defensive response (Kyrou and Tsigos, 2007). Thus, given that, both stress and ghrelin favor the use of carbohydrate substrates, which are rapidly oxidized, and the enhanced secretion of ghrelin following stress, it is likely that ghrelin contributes to the stress-induced metabolic switch that favors carbohydrate utilization and the accumulation of fat stores. Indeed, elevated ghrelin levels following chronic exposure to social stress increase caloric intake, preserve fat stores and stimulate body weight gain in mice, while under the same social stress paradigm these metabolic responses are attenuated or absent in GHS-R1a knockout mice and following pharmacological blockade of the GHS-R1a receptor (Patterson et al., 2013). Thus, enhanced ghrelin levels subsequently increase the hedonic and rewarding value of food, which further stimulates the intake of palatable and caloric dense “comfort” foods, which elicits activation of central reward pathways and increases dopamine signaling. This may mediate metabolic adaptations in response to the psychosocial stressors to meet the higher energetic demands and may act to reduce the detrimental effects of these stressors to promote survival and may ultimately protect against anxiety- and depression-like behaviors (Schellekens et al., 2012b).

However, chronic stress can lead to metabolic dysfunction and aberrant ghrelin signaling, which may ultimately lead to obesity and a downregulated reward signaling, which may be causal to the increased sensitivity for the development of psychiatric disorders including depression and anxiety. Thus, ghrelin and its receptor, the GHS-R1a receptor, likely represent key metabolic regulators required to cope with stress and to prevent excessive anxiety under conditions of chronic stress (Patterson et al., 2010). However, prolonged exposure to stress and stress-induced elevations in ghrelinergic signaling may unmask negative metabolic consequences, leading to long-lasting metabolic dysfunction and ultimately obesity as well as an associated sensitivity to the stress-induced psychiatric disorders of anxiety and depression (Patterson et al., 2010; Schellekens et al., 2012b).

In conclusion, it is clear that classical feeding peptides, including ghrelin, are strongly linked in the regulation of appetite and food intake, the rewarding and motivational drive to eat and the ability to cope with stress. The precise molecular mechanism linking ghrelin in appetite, reward and stress are not fully elucidated but it is clear that connections to DA neurons in the mesolimbic circuitry system are important. We hypothesize that direct interactions of the GHS-R1a receptor with other GPCRs, which have recently been described, are poised to play a key role in the association of these ghrelin-mediated physiological responses in reward signaling and stress. This review will further discuss the potential dynamic role for GHS-R1a receptor dimerization in the regulation of ghrelin-specific functions within stress response and food intake.

## Modulation of G-protein coupled receptors via oligomerization

The GHS-R1a receptor belongs to the class A GPCRs, which are seven transmembrane domain proteins in the plasma membrane of cells. Conformational changes of the receptor are mediated via interactions of the GPCR with extracellular small-molecule ligands, which subsequently results in interaction and activation of hetero-trimeric intracellular G proteins, which further transduce the downstream signaling events necessary for the cell to respond to changes in the environment (Pierce et al., 2002). GPCRs represent the largest family of proteins (>800 members) in the human genome and are involved in nearly all physiological processes, mediating signal transductions across the cellular membrane. Many GPCRs have been implicated in the pathogenesis of human disease and thus, represent attractive targets for the development of drugs in the pharmaceutical industry. However, GPCR-targeting drugs are often associated with side effects due to non-specific activation of other GPCR and non-GPCR targets and novel pharmacotherapies with enhanced specificity are urgently sought after.

The concept of the monomeric existence and functioning of GPCRs, including the GHS-R1a receptor, is firmly established in literature and has been confirmed by several lines of evidence from recent studies (Whorton et al., 2007, 2008; Rasmussen et al., 2011; Damian et al., 2012). However, a large accumulating body of biochemical and biophysical work now supports the notion that GPCRs, including class A GPCRs, to which the GHS-R1a receptor also belongs, do not exclusively exist as monomeric entities, but extensively crosstalk with each other and form physiologically relevant oligomers (for review see George et al., 2002; Franco et al., 2008; Birdsall, 2010; Rozenfeld and Devi, 2010; Gonzalez-Maeso, 2011; Kamal and Jockers, 2011; Rozenfeld and Devi, 2011; Goddard and Watts, 2012; Herrick-Davis, 2013; Milligan, 2013; Ward et al., 2013). Indeed, many GPCRs have been recently found to function as oligomeric complexes, whereby receptors of the same or different families combine to generate homo- or heterodimers or even higher-structure multimeric complexes (Gupta et al., 2010; Rivero-Muller et al., 2010; Teitler and Klein, 2012; Jastrzebska et al., 2013). Moreover, elaborate methods such as fluorescence imaging studies, including fluorescence recovery after photobleaching (FRAP) and total internal reflection fluorescence imaging (TIRF) of single molecules, as well as intracellular trafficking experiments, have been explored to confirm both monomeric GPCRs as well as stable and transient GPCR oligomers (Dorsch et al., 2009; Hern et al., 2010; Calebiro et al., 2013; Gavalas et al., 2013). Thus, GPCR oligomerization can now be considered as a fundamental process in receptor signaling. These GPCR oligomers exhibit unique pharmacological, biochemical and functional characteristics including specific signaling cascades, binding cooperativity, altered receptor internalization as well as changes in recycling properties (Figure 1) (Hebert and Bouvier, 1998; Terrillon and Bouvier, 2004; Springael et al., 2005; Urizar et al., 2005; Kent et al., 2007; Smith and Milligan, 2010; Urizar et al., 2011; de Poorter et al., 2013). Dimerization of GPCRs may explain the high constitutive activity of some GPCRs and heterodimerization may be obligatory for GPCR maturation and trafficking from the endoplasmic reticulum (ER) to the cell surface to ensure full receptor functionality [Figure 1(1)], as has been seen for the GABA_B_ receptors, which is a class C GPCR (Kaupmann et al., 1998; Cryan and Kaupmann, 2005). The metabotropic GABA_B_ receptor requires both GABA_B_R1 and GABA_B_R2 receptors for functional receptor expression on the cell membrane (White et al., 1998; Galvez et al., 2000, 2001; Duthey et al., 2002). Heterologous expression of the GABA_B_R1 subunit without the GABA_B_R2 leads to retention of the subunit in the ER, while the GABA_B_R2 subunit alone is expressed on the membrane but not functional. Heterodimerization of both GABA subunits causes masking of a retention signal and functional receptor expression (Margeta-Mitrovic et al., 2000). Similarly, homodimerization of two β2-adrenergic receptor (β2-AR) subunits is critical for cell surface expression (Salahpour et al., 2004) and the α1δ-AR is only fully functional and rescued from the ER when heterodimerized with a α1β-ARs or β2-AR subunit (Uberti et al., 2005; Hague et al., 2006). Secondly, receptor dimerization may be dynamically regulated following ligand-mediated receptor activation [Figure 1(2)] and specific ligand binding may promote or inhibit dimerization (Horvat et al., 2001; Patel et al., 2002). Alternatively, dimerization may alter the ligand-binding properties of the receptor complex. In addition, heterodimerization can have consequences on allosteric potentiating or attenuating of downstream signaling [Figure 1(3)] as well as influencing positive or negative co-operativity of ligand binding (Terrillon and Bouvier, 2004). Moreover, heterodimerization may have the potential to change G-protein selectivity, alter G-protein coupling [Figure 1(4)] and alter subsequent downstream signaling and GPCR specificity (Rozenfeld and Devi, 2011). Interestingly, oligomerized receptor complexes have been shown to couple to multiple G-proteins, depending on their cellular environment, thereby conferring the ability to mediate different intracellular responses to the same ligand (Rashid et al., 2004). Heterodimerization may also promote co-internalization of two receptors after the stimulation of only one protomer. Alternatively, differential desensitization and β-arrestin recruitment may occur and the presence of a protomer that is resistant to agonist-promoted endocytosis, within a heterodimer, can inhibit the internalization of the complex [Figure 1(5)]. Heterodimer GPCR expression will likely not be the same in all tissue as not all GPCRs are equally expressed across various cell types. Therefore, tissue specific expression of GPCRs will directly regulate dimerization and may explain the tissue specificity of current GPCR-targeting drugs. In addition, heterodimerization of GPCRs will likely be different under pathological human conditions, including obesity and depression, which may be exploited in the development of novel ligands with increased specificity and selectivity.

However, current evidence for GPCR interactions are limited by the fact that the vast majority of studies to date have been performed in model systems only, which may explain why GPCR oligomerization is still met with a certain degree of controversy. In addition, during the interpretation of results it has proved to be very difficult to exclude cross-talk. Therefore, conclusions on GPCR oligomerization should be taken with caution. Although in-depth discussion of the limitations of previous studies is beyond the scope of this manuscript, several other reviews discussing this topic exists (Ferre et al., 2009; Birdsall, 2010; Teitler and Klein, 2012; Milligan, 2013).

While the concept of GPCR is certainly interesting, further validation at the physiological level is required to convincingly show that GPCRs indeed form physiologically relevant dimers. However, demonstrating dimerization in native tissue will be challenging as GPCRs are not always very highly expressed endogenously and many GPCRs, including the GHS-R1a receptor, are often associated with high constitutive activity (Holst et al., 2003; Petersen et al., 2009; Els et al., 2010). Previously, the acceptance of the functioning of GPCR dimers *in vivo* was dependent on co-expression of both protomers for functionality or the abolishment of heteromeric receptor function following genetic deletion of one protomer (Pin et al., 2007; Ferre et al., 2009; Teitler and Klein, 2012). Moreover, the complex and dynamic nature of GPCR dimerization needs to be considered, as dimerization has been suggested to be both reversible as well as ligand and cell type dependent (Smith and Milligan, 2010). Thus, additional evidence demonstrating dimers *in vivo* is warranted as well as more defined criteria. The advancement of the field will be complicated by the fact that many class A GPCRs, including the GHS-R1a receptor are most likely functional as both monomeric units as well as in heterodimers or oligomeric complexes. The next steps forward for the field will include the development and utilization of advanced techniques with the capability to extrapolate dimerization from overexpressing model systems to wild-type GPCRs expressed in native tissues using for example fluorescent ligands in FRET-based binding assays (Albizu et al., 2010; Cottet et al., 2012, 2013).

Nevertheless, the implications of enhanced diversity of GPCRs pharmacology following GPCR oligomerization fundamentally changes our current knowledge on the structure, activation and desensitization processes of GPCRs. Ultimately, GPCR dimerization is poised to have a dramatic impact on drug development and screening as it opens up new avenues for the development of potential novel therapeutics targeting GPCRs, including the GHS-R1a receptor (George et al., 2002; Waldhoer et al., 2005; Panetta and Greenwood, 2008; Casado et al., 2009; Valant et al., 2009; Rozenfeld and Devi, 2010, 2011).

## GHS-R1a receptor dimerization and implications in stress and reward signaling

Evidence is accumulating demonstrating dimerization of the GHS-R1a receptor (Chan and Cheng, 2004; Holst et al., 2005; Jiang et al., 2006; Takahashi et al., 2006; Chu et al., 2007; Leung et al., 2007; Chow et al., 2008, 2012; Rediger et al., 2009, 2011; Kern et al., 2012; Park et al., 2012; Schellekens et al., 2013b). Dimerization of the GHS-R1a receptor into homo- and/or heterodimers has the potential to significantly alter downstream signaling. Indeed, evidence from mostly heterologous expression systems and also some native cells has demonstrated that the GHS-R1a receptor can both traffic and signal as higher-order oligomeric-complexes dimerized with additional GPCRs involved in appetite regulation, food reward and stress (Jiang et al., 2006; Rediger et al., 2009, 2011; Kern et al., 2012; Schellekens et al., 2013b). This promiscuous dimerization of the GHS-R1a receptor may function to fine-tune receptor mediated activity via differential downstream signaling and altered GHS-R1a receptor trafficking. This paragraph describes GHS-R1a receptor dimers known to date and discusses their potential functional significance. GHS-R1a dimerization is poised to impact on the regulation of food intake, hedonic appetite signaling and stress-induced food intake, in which the ghrelinergic system has been shown to play a major role.

### Homodimers and heterodimers of the GHS-R1a receptor

Recent research has identified that the GHS-R1a receptor also has the ability to form homodimers as well as to dimerize with other GPCRs, forming heterodimers (Table 1). In 2005, Holst and et al. were the first to propose a model in which the GHS-R1a receptor functions as a homodimer (Holst et al., 2005). In this study it was shown that coadministration of a non-endogenous agonist can act as a neutral (MK-677), positive (L-692,429), or negative (GHRP-6) modulator of the GHS-R1a receptor in the presence of the endogenous GHS-R1a agonist ghrelin. The hypothesized homodimeric model of the GHS-R1a receptor was supported by the potentiated response of ghrelin upon binding of growth hormone-releasing hormone (GHRH) to the GHS-R1a receptor (Casanueva et al., 2008). Co-administration of GHRH dose-dependently potentiated ghrelin-induced cellular calcium mobilization and inositol phosphate turnover via Gq-associated signal transduction without competing with ghrelin for binding but enhancing ghrelin's binding capacity. This positive binding co-operativity was suggested to occur following the interaction of GHRH on an allosteric binding site of the GHS-R1a receptor, acting as a co-agonist in presence of the endogenous ligand ghrelin. This allows for an increased affinity of ghrelin for the GHS-R1a receptor and the simultaneous binding of two ghrelin molecules, one to each GHS-R1a monomer subunit within the homodimer, while in the absence of ghrelin GHRH binds to the orthosteric binding site of GHS-R1a (Casanueva et al., 2008). However, it cannot be ruled out that a direct interaction between the GHS-R1a and GHRH receptor is responsible for the synergistic interaction, which is reinforced by the observed potentiation of GHRH-mediated cAMP production when the GHS-R1a receptor is co-expressed (Cunha and Mayo, 2002). The homodimeric GHS-R1a model was confirmed in human embryonic kidney cells (Hek), using bioluminescence resonance energy transfer (BRET) and co-immunoprecipitation (Leung et al., 2007). Further evidence for a direct physical interaction of two GHS-R1a subunits in a homodimeric complex or a heterodimer between the GHS-R1a and the GHRH receptor and the existence of such dimers *in vivo* is warranted.

**Table 1:** **Homo- and heterodimerization of the GHS-R1a receptor**.

**Dimer**	**Physical interaction *in vitro***	**Cell lines**	**Physical interaction *in/ex vivo***	**Functional interaction *in vivo***	**References**
GHS-R1a/GHS-R1a	Binding and signal transduction assays demonstrate allosteric modulation of ghrelin signaling: calcium mobilization, inositol phosphate turnover, CRE and SRE transcription assay, β-arrestin mobilization, BRET, co-IP	Hek, COS-7	nd	nd	Holst et al., 2005; Leung et al., 2007
GHS-R1a/GHS-R1b	Subcellular co-localization using immunocytochemistry; BRET; ghrelin binding assay; cell surface expression ELISA; receptor downstream signaling; co-IP	Hek, CHO	nd	nd	Chan and Cheng, 2004; Chu et al., 2007; Leung et al., 2007; Chow et al., 2012; Mary et al., 2013
GHS-R1a/EP3-1	Co-IP; BRET	Hek	nd	nd	Chow et al., 2008
GHS-R1a/IP	Co-IP; BRET	Hek	nd	nd	Chow et al., 2008
GHS-R1a/TPα	Co-IP; BRET	Hek	nd	nd	Chow et al., 2008
GHS-R1a/SST5	Glucose-stimulated insulin secretion assay; tr-FRET; BRET; downstream Gq (calcium assay) and Gs (cAMP) signaling	Hek, INS-1SJ	nd	nd	Park et al., 2012
GHS-R1b/NTS_1_	Co-IP; Co-localization and receptor trafficking	Cos-7, LC319	nd	nd	Takahashi et al., 2006
GHS-R1a/MC_3_	FRET; ELISA; Co-localization; binding assay; receptor trafficking and downstream Gq (NFAT-luciferase reporter or calcium assay) and Gs (cAMP) signaling	Hek, COS-7	Co-expression of MC_3_ with lacZ-immunoreactive cells, representing GHS-R1a in Arc of GHS-R1a knock-out mice	nd	Rediger et al., 2009, 2011; Schellekens et al., 2013b
GHS-R1a/D_1_	Co-IP; Co-localization; BRET; receptor trafficking and downstream Gq (calcium assay) and Gs (cAMP) signaling	Hek, SK-N-SH	Co-expression of D_1_ with GHS-R1a in the VTA of GHSR-IRES-tauGFP knock-in homozygous mice	nd	Jiang et al., 2006; Schellekens et al., 2013b
GHS-R1a/D_2_	Co-localization; downstream signaling Gq and Gβγ subunit of Gi (calcium mobilization assay and imaging); tr-FRET	Hek, SH-SY5Y, Primary hypothalamic neurons	Co-expression of D_2_/GHS-R1a in hippocampus, striatum and hypothalamus of GHSR-IRES-tauGFP knock-in homozygous mice; Tr-FRET of striatum and hypothalamic tissue in wt and GHSR^−/−^ mice	Allosteric function for GHS-R1a on D_2_-mediated inhibition of food intake. D_2_ agonist cabergoline reduces food intake in mice, which is absent when GHS-R1a is pharmacologically blocked or knocked out in GHS-R1a^−/−^ mice	Kern et al., 2012
GHS-R1a/5-HT_2C_	Co-localization; receptor trafficking and downstream Gq (calcium assay) signaling	Hek	nd	nd	Schellekens et al., 2013b

Abbreviations: BRET, bioluminescence resonance energy transfer; co-IP, co-immunoprecipitation; CRE, cAMP-responsive element; ELISA, Enzyme-linked immunosorbent assay; D_1/2_, dopamine receptor 1/2; GHS-R, growth hormone secretagogue receptor; IP, prostaglandin (prostacyclin) receptor; EP, prostanoid receptor; MC_3_, melanocortin receptor 3; nd, not done; NFAT, nuclear factor of activated T cells; NTS1, neurotensin 1 receptor; SRE, serum-responsive element; TPα, thromboxane A2; tr-FRET, time-resolved fluorescence resonance energy transfer.

In addition, evidence is accumulating suggesting that the GHS-R1a receptor has the ability to form a heterodimer with its non-signaling truncated splice variant, the GHS-R1b receptor. The GHS-R1b receptor is a splice variant with only 5 transmembrane domains, lacking the last 2 transmembrane domains typical for GPCRs, and is primarily localized in the ER (Chan and Cheng, 2004; Schellekens et al., 2010; Chow et al., 2012). This dimerization has been shown to attenuate the GHS-R1a/GHS-R1b receptor pair in the in the ER, which consequently reduces ghrelin responsiveness and suggests the GHS-R1b receptor to act as a dominant-negative mutant of the full-length GHS-R1a receptor (Chan and Cheng, 2004; Chu et al., 2007; Leung et al., 2007; Chow et al., 2012). The existence of the GHS-R1a/GHS-R1b heterodimer was reinforced using BRET and in co-immunoprecipitation experiments and a decreased cell surface expression and decreased constitutive GHS-R1a receptor activity were observed with increasing expression of GHS-R1b (Leung et al., 2007; Chow et al., 2012). Interestingly, dimerization of the mu-opioid with truncated splice variants was recently shown to increase total membrane receptor expression (Pasternak, 2013; Xu et al., 2013). In addition, a recent study using purified GHS-R monomers and dimers reconstituted into lipid discs revealed that the dominant negative effect of the truncated GHS-R1b receptor is exerted via a conformational restriction of the full-length GHS-R1a protein, which blocks subsequent G protein activation and β-arrestin recruitment. This clearly demonstrates heteromer-directed selectivity whereby the specific dimerization impacts on the functional and structural behavior of the ghrelin receptor (Mary et al., 2013).

Moreover, the GHS-R1a receptor was found to dimerize with members of the prostanoid receptor family, which are involved in modulating vascular activity and inflammatory responses (Chow et al., 2008). Heterodimers of the GHS-R1a receptor with the vasodilator prostacyclin (IP) receptor, the vasoconstrictor prostaglandin E2 receptor subtype EP3-I (EP3-1) and the thromboxane A2 (TPα) receptor were demonstrated using co-immunoprecipitations and BRET following transient co-expression in Hek cells. Decreased GHS-R1a receptor expression, increase intracellular GHS-R1a receptor localization and attenuated constitutive GHS-R1a receptor activation, were observed upon co-expression of prostanoid receptors, suggesting a dynamic regulation of GHS-R1a receptor activity under conditions of increased prostanoid receptors expression, including vascular inflammation and in atherosclerotic plaques.

Furthermore, the GHS-R1b receptor was shown to physically interact with the neurotensin receptor 1 (NTS1) and this GHS-R1b/NTS1 heterodimer was suggested to play a role in the autocrine growth-promoting pathway of non–small cell lung cancers (Takahashi et al., 2006).

Finally, a recent study indicates that the GHS-R1a receptor also interacts with the somatostatin receptor family, specifically with the somatostatin receptor-5 (SST5) (Park et al., 2012). Constitutive formation of a GHS-R1a/SST5 heterodimer was shown using time-resolved FRET and BRET assays, in which endogenous ghrelin rather than SST, suppressed glucose-stimulated insulin secretion (GSIS) from pancreatic β-cells. This was shown to involve a noncanonical ghrelin receptor (GHS-R1a)–G-protein coupling to Gαi/o and subsequent increased cAMP production, instead of coupling to the Gαq11 subunit. Moreover, the formation of GHS-R1a/SST5 heterodimer demonstrated to be dependent on a high ratio of ghrelin to SST, suggesting a dependence on energy balance, with enhanced dimer formation following fasting-induced peak ghrelin levels. This model predicts that under conditions of low energy balance the formation of a physiologically relevant GHS-R1a/SST5 heterodimer establishes an inhibitory tone on β-cells via ghrelin signaling. This may potentially explain the differential regulation of islet function by ghrelin and SST and reinforces the potential dynamic nature of GPCR dimerization.

### A GHS-R1a/MC_3_ receptor heterodimer in appetite signaling

Evidence for other GHS-R1a heterodimerization pairs is accumulating and to date heterodimerization of the GHS-R1a receptor with several other hypothalamic GPCRs involved in appetite signaling has been demonstrated (Rediger et al., 2009, 2011; Schellekens et al., 2013b). Evidence for a physical interaction between the MC_3_ and GHS-R1a receptor was demonstrated, using enzyme-linked immuno sorbent assay (ELISA) and fluorescence resonance energy transfer (FRET) approaches in Hek cells (Rediger et al., 2009). A more recent study, confirmed dimerization of the GHS-R1a receptor in the ARC of the hypothalamus with the MC_3_ receptor (Rediger et al., 2011), which is an important downstream signaling receptor in the homeostatic control of food intake and energy balance (Cone et al., 1996; Kishi et al., 2003; Adan et al., 2006; Yang, 2011). The study demonstrated a mutual signaling interference upon receptor dimerization. Ghrelin-induced Gq-mediated GHS-R1a receptor signaling was significantly reduced upon co-expression of the MC3 receptor in COS-7 cells, while MC_3_-mediated cAMP signaling upon administration of alpha-MSH was enhanced in Hek cells co-expressing both receptors (Rediger et al., 2011). In addition, a strong attenuation of GHS-R1a mediated downstream signaling was shown upon co-expression of both the MC_3_ and the GHS-R1a receptors in Hek cells, which has been suggested to be most likely due to an attenuation of the dimer pair in the cytosol of Hek cells (Rediger et al., 2009; Schellekens et al., 2013b). However, no decrease of membrane receptor expression was observed when both GHS-R1a and MC_3_ were expressed in COS-7 cells, as investigated by ELISA and binding studies, which may indicate cell-specific effects (Rediger et al., 2011).

### Dimerization of the GHS-R1a receptor with dopamine D_1_ and D_2_ receptors

Considering the involvement of ghrelin signaling in other physiological pathways besides appetite, evidence of heterodimers of the GHS-R1a with extra-hypothalamic GPCRs involved in hedonic and rewarding aspects of food intake may be able to explain crosstalk between the ghrelinergic and mesolimbic dopaminergic systems. In line with this concept, is the recent discovery that the GHS-R1a receptor can form dimers with dopamine D_1_ and D_2_ receptors (Jiang et al., 2006; Kern et al., 2012). The formation of GHS-R1a/D_1_ or GHS-R1a/D_2_ receptor heterodimers are poised to have important functional consequences for the role of ghrelin in the regulation of rewarding and motivational eating behavior. The GHS-R1a receptor was first reported to be able to dimerize with the dopamine D_1_ receptor (Jiang et al., 2006). In this study, Hek cells expressing both the GHS-R1a and the D_1_ receptor displayed a ghrelin-mediated potentiation of dopamine induced c-AMP accumulation in a GHS-R dependent manner. Additional evidence for a dimerization of the GHS-R1a receptor with the D_1_ receptor was recently demonstrated by the attenuation of GHS-R1a-mediated calcium signaling in Hek cells coexpressing both receptors (Schellekens et al., 2013b). Together, this may suggest a dimer-induced switch in GHS-R1a receptor G-protein coupling from Gq to Gs mediated signaling, which has been previously suggested for neuronal GHS-R1a receptors expressed in NPY cells of the ARC (Kohno et al., 2003). In addition, co-internalization of the GHS-R1a/D_1_ receptor pair was also demonstrated following agonist treatment, further supporting the existence of a GHS-R1/D_1_ heterodimer (Schellekens et al., 2013b).

Recently, the D_2_ receptor has also been found to dimerize with the GHS-R1a receptor in hypothalamic neurons and to alter canonical D_2_ signal transduction resulting in dopamine-induced calcium mobilization (Kern et al., 2012). Coexpression of the D_2_ and the GHS-R1a receptor in mouse neuronal hypothalamic neurons was shown as well as in hippocampal and striatal neurons. In addition, a D_2_-dependent increase in calcium was observed in SH-SY5Y neuroblastoma cells and Hek cells overexpressing both receptors as well as in primary hypothalamic cells using calcium mobilization assay or calcium imaging to monitor calcium influxes in live cells. A significant increase in intracellular calcium was observed following treatment with dopamine or a selective D_2_ agonist, quinpirole, only in cells co-expressing the Gi/o-coupled D_2_ receptor and the Gq-coupled GHS-R1a receptor. Moreover, the dopamine-induced calcium mobilization was demonstrated to be independent of GHS-R1a-Gq mediated signaling or basal GHS-R1a receptor activity, as Gq siRNA was unable to inhibit dopamine-induced calcium release in Hek cells, but the involvement of Gβγ subunits in the calcium mobilization from intracellular stores and Gβγ-mediated activation of PLC was shown. Finally, the functional interaction between the D_2_ and GHS-R1a receptor was demonstrated *in vitro* in Hek cells as well as *in vivo* in native hypothalamic mouse tissue using time resolved fluorescence energy transfer (tr-FRET) and confocal FRET. This study by Kern and colleagues is one of the very few studies to extrapolate the evidence from overexpressing model system to naïve tissue with endogenously expressed GHS-R1a receptor. Most importantly, the GHS-R1a/D_2_ heterodimer was shown to allosterically modify D_2_-mediated calcium mobilization, as the modification of D_2_-mediated signaling was observed in the absence of the endogenous GHS-R1a ligand ghrelin, but was blocked by both D_2_ and GHS-R1a antagonism. This highlights a potential function of the GHS-R1a receptor expressed in areas of the brain without ghrelin immunoreactivity and which are considered inaccessible to peripherally produced ghrelin. Finally, the functional relevance of the GHS-R1a/D_2_ dimer was investigated and it was demonstrated that D_2_/GHS-R1a pairing attenuates food intake (Kern et al., 2012). Evidence for the allosteric function for GHS-R1a on D_2_-mediated inhibition of food intake was shown using the D_2_ agonist cabergoline, which reduced food intake in mice. The cabergoline-induced anorexia did not require ghrelin but was dependent on GHS-R1a signaling and the GHS-R1a-D_2_ interaction as it was blocked when the GHS-R1a receptor was pharmacologically inhibited or knocked out in GHS-R1a^−/−^ mice. Ghrelin has previously been suggested to interact with dopamine and to modulate appetitive behavior within the dopaminergic reward circuitry (Abizaid et al., 2006; Abizaid, 2009; Weinberg et al., 2011). Indeed, the ghrelinergic system has been shown to alter the rewarding value of foods and to elicit a preference for palatable foods rich in sugar or fats via modulation of dopamine release (Egecioglu et al., 2010). This may suggest a role for GHS-R1a receptor dimerization in the regulation of rewarding qualities of food, independent of homeostatic regulation of food intake, and the function of GHS-R1a heterodimers in the hedonic appetite signaling warrants further investigation.

### Evidence for a heterodimer between the GHS-R1a receptor and the 5-HT_2C_ receptor

Finally, in a recent study evidence was presented for a novel heterodimer between the GHS-R1a receptor and the 5-HT_2C_ receptor (Schellekens et al., 2013b). Here it was shown that GHS-R1a-mediated calcium mobilization following ghrelin or MK0677 treatment, was attenuated following interaction with the fully active unedited form of the 5-HT_2C_ receptor but not following co-expression of a partially edited isoform of the 5-HT_2C_ receptor. Editing of the 5-HT_2C_ receptor has been demonstrated to lead to a decreased receptor functioning (Burns et al., 1997; Niswender et al., 1999; Berg et al., 2008; Olaghere da Silva et al., 2010; Schellekens et al., 2012a). This may suggest the possibility that alteration of the 5-HT_2C_ receptor editing profile can equally impact on GHS-R1a receptor signaling *in vivo*. The attenuated GHS-R1a receptor signaling was completely restored following pharmacological blockade of the 5-HT_2C_ receptor. Moreover, ligand-mediated co-internalization of the GHS-R1a/5-HT_2C_ receptor pair was demonstrated in Hek cells generated to express the receptor pair. Further evidence for a GHS-R1a/5-HT_2C_ receptor dimer was shown when a potent specific inverse agonist of the GHS-R1a receptor, [D-Arg1, D-Phe5,D-Trp7,9, Leu11]-substance P, was able to increase signaling in heterologous cells co-expressing the GHS-R1a/5-HT_2C_ receptor dimer following serotonin (5-HT, 5-hydroxytryptamine) treatment, while no potentiation of Gq-mediated calcium mobilization was observed in cells solely expressing the 5-HT_2C_ receptor. The inverse GHS-R1a agonist suppresses ligand-independent basal activity of the GHS-R1a receptor (Holst et al., 2003) and is known to increase GHS-R1a receptor membrane expression (Liu et al., 2007). This data demonstrates a co-recruitment of the 5-HT_2C_ receptor with the GHS-R1a receptor to the cellular membrane, upon treatment with the inverse GHS-R1a receptor agonist, indicating a direct physical interaction. Together, this suggests a potential novel mechanism of 5-HT_2C_ mediated attenuation of the GHS-R1a receptor. Both the GHS-R1a receptor and the 5-HT_2C_ receptor are involved in feeding behavior. Activation of the 5-HT_2C_ receptor signaling mediates hypophagia (Lam et al., 2008). Interestingly the 5-HT_2C_ receptor also mediates anxiety like behavior (Heisler et al., 2007b). It is therefore tempting to speculate that GHS-R1a/5-HT_2C_ receptor heterodimerization may play a role in homeostatic appetite signaling and in stress-induced food intake. A recent study demonstrated an attenuation of ghrelin's orexigenic effect following direct PVN administration of 5-HT in rats, which supports a potential significant role for the GHS-R1a/5-HT_2C_ receptor dimer pair in appetite regulation *in vivo* (Currie et al., 2010). Further investigations into the extent and functional relevance of GHS-R1a/5-HT_2C_ dimerization *in vivo* are now warranted.

## Conclusion

Obesity is a growing concern increasing in prevalence worldwide. In addition, humans are increasingly faced with excessive psychological stress in modern day society, which appears to have synergistic effects with the obesity epidemic. The orexigenic gastric-derived hormone ghrelin plays a key role in the homeostatic control of appetite, rewarding and motivational aspects of food intake as well as stress-induced food reward behaviors, linking eating behavior with both stress and hedonic signaling (for review see Chuang and Zigman, 2010; Diz-Chaves, 2011; Perello and Zigman, 2012; Schellekens et al., 2012b). Ghrelin signals via the peripheral and central expressed GHS-R1a receptor, which has recently shown to form heterodimers with several additional GPCRs involved in appetite signaling and reward, including the truncated GHS-R1b receptor, the MC_3_ receptor, D_1_ and D_2_ receptors, and more recently, the 5-HT_2C_ receptor (Jiang et al., 2006; Rediger et al., 2009, 2011; Kern et al., 2012; Schellekens et al., 2013b). Heterodimerization of the GHS-R1a receptor may function to fine-tune GHS-R1a mediated signaling and could potentially explain crosstalk between ghrelin and monoaminergic neurotransmission in the brain. Heterodimerization may regulate GHS-R1a receptor function via stabilization of specific receptor conformations, altering preferential G-protein coupling and lead to heteromer-specific signal transduction and receptor trafficking.

Overall, GHS-R1a heterodimerization may modulate specific signaling pathways and GHS-R1a receptor functionalities or serve as an allosteric mechanism able to regulate signaling pathways of the other receptor, independently of ghrelin binding. The latter has been demonstrated recently for the GHS-R1a/D_2_ heterodimer by the observation that GHS-R1a receptor expression alone was able to allosterically mediate D_2_ receptor signaling (Kern et al., 2012). In addition, this study also demonstrated that dopamine-mediated anorexia was inhibited following the pharmacological blockade of the GHS-R1a receptor. Interestingly, D_2_ receptor mutations have demonstrated to attenuate dopamine signaling and to be associated with human obesity (Tataranni et al., 2001). Moreover, since central ghrelin signaling has been shown to be involved in the control of reward seeking behaviors for food, alcohol, and drugs of abuse by modulating mesolimbic dopaminergic reward signaling, the existence of GHS-R1a/D_2_ heterodimers would offer a potential mechanism to explain the effects of ghrelin on reward. Moreover, dopamine signaling has been shown to be crucial for ghrelin-mediated increase in food reward. Thus, the GHS-R1a/D_2_ dimer would represent a new molecular target and rational for the development of ghrelin-based therapeutic interventions in conditions associated with abnormal reward-seeking behavior. Moreover, circulating ghrelin levels are enhanced following acute stress and interactions of the GHS-R1a receptor with other GPCRs mediating the rewarding and motivational drive to eat, such as the D_1_ and D_2_ receptor, may activate pathways necessary to cope with stress.

The stress-induced increase in ghrelin has been suggested to be able to reduce anxiety under conditions of acute stress following the stimulation of the HPA and the neurobiology of stress and appetite regulation overlap significantly. The 5-HT_2C_ receptor is expressed in the ARC of the hypothalamus mediating hypophagia (Lam et al., 2008) as well as on CRF neurons in the PVN and similar to ghrelin, 5-HT_2C_ receptor activation here, mediates secretion of CRF (Heisler et al., 2007a). Moreover, 5-HT_2C_ receptor knockdown decreases anxiety-like behavior (Heisler et al., 2007b), while mice with genetic ablation of ghrelin exhibit a more anxious phenotype (Spencer et al., 2012). Together this may suggest a functional role for the recently identified GHS-R1a/5-HT_2C_ dimer in HPA axis activation, which warrants further investigations.

Targeting GHS-R1a receptor dimers within the metabolic regulation of food intake and food reward is poised to impact on the dopamine-related reward systems and stress response and therefore not only provides exiting opportunities for future pharmacotherapies in the treatment of metabolic and eating disorders, but also in psychiatric disorders with dysregulated DA signaling. The recent findings demonstrating heterodimerization of the GHS-R1a under certain conditions and reinforces the concept that GPCR signaling is most likely a dynamic process, which is dependent on physiological context. Understanding the contributions of the interactions between the serotinergic and ghrelinergic system on the dopaminergic output following stress and maladaptive food intake and the role of the recently identified GHS-R1a heterodimer complexes herein, will increase our knowledge of the pathology of obesity and anxiety and its bidirectional relationship. While the GHS-R1a receptor is expressed ubiquitously in the brain at multiple sites involved in both food intake behavior and stress, the MC_3_, 5-HT_2C_, D_1_ and D_2_ receptors have a more localized expression. For example the MC_3_ is expressed in the hypothalamus only, which means that the GHS-R1a/MC_3_ dimer potentially only has an impact on homeostatic regulation of appetite. The exact central locations of GHS-R1a complex formation remain to be determined.

Noteworthy, evidence supporting a physiologically relevant interaction and physiological role for GHS-R1a heterodimers is limited. It has been notoriously difficult to establish the existence of the vast majority of GPCR dimers *in vivo* due to the experimental challenge of differentiating a direct physical interaction from receptor crosstalk and this remain somewhat controversial. Nevertheless, the conceptually important premise of promiscuous GHS-R1a heterodimerization confers unique pharmacological and functional properties to the receptor, significantly enhancing the pharmacological diversity and with it the potential for novel therapies. Additional research is warranted to further investigate the physiological relevance of GHS-R1a dimers in metabolic eating disorders, such as obesity, and under conditions of stress. Further studies are required to determine whether the dimerization in the *in vitro* biochemical assays translates to the functionally relevant dimers *in vivo*. Nevertheless, the oligomerization characteristic of GPCRs, including the GHS-R1a receptor, creates a broader regulatory complexity and has therapeutically important implication as heterodimers provide novel pharmacological targets and thus may be exploited to create more specific therapeutic drugs (Prinster et al., 2005). Thus, although more evidence and functional relevance for GPCR dimerization *in vivo* as well as methods to deal with the most likely dynamic nature of physical receptor interaction is warranted, the achievements of the last few years on GPCR dimerization provide the foundation of what promises to be very exciting times for GPCR-targeted molecular pharmacology. In addition, the field of heterodimer specific pharmacology and drug discovery is set to change significantly with these new findings.

### Conflict of interest statement

The authors declare that the research was conducted in the absence of any commercial or financial relationships that could be construed as a potential conflict of interest.

## Abbreviations

ACTH: adrenocorticotropic hormone
AgRP: agouti-related peptide
ARC: arcuate nucleus
BRET: bioluminescence resonance energy transfer
CART: cocaine- and amphetamine-regulated transcript
CCK: cholecystokinin
CPP: conditioned place preference
CSDS: chronic social defeat stress
CRF: corticotrophin-releasing factor
DA: dopamine
GH: growth hormone
GHRH: growth hormone-releasing hormone
D_1_: dopamine 1 receptor
D_2_: dopamine 2 receptor
FRAP: fluorescence recovery after photobleaching
GHS-R1a: growth-hormone secretagogue receptor
GOAT: ghrelin O-acyl transferase
GPCR: G-protein coupled receptors
Hek: human embryonic kidney cells
HPA: hypothalamic-pituitary-adrenal
MC3: melanocortin 3 receptor
NAcc: nucleus accumbens
NPY: neuropeptide Y
NTS: nucleus of the solitary tract (nucleus tractus solitarii)
PVN: paraventricular nucleus
POMC: proopiomelanocortin
SN: substantia nigra
TIRF: total internal reflection fluorescence imaging
UPC2/3: uncoupling proteins 2 and 3
VTA: ventral tegmentum
5-HT: serotonin (5-hydroxytryptamine)
5-HT_2C_: serotonin 2C receptor
6-OHDA: 6-hydroxydopamine.
